# The effect of socio-demographic factors on mental health and addiction high-cost use: a retrospective, population-based study in Saskatchewan

**DOI:** 10.17269/s41997-018-0101-2

**Published:** 2018-06-28

**Authors:** Maureen Anderson, Crawford W. Revie, Jacqueline M. Quail, Walter Wodchis, Claire de Oliveira, Meriç Osman, Marilyn Baetz, J. McClure, Henrik Stryhn, David Buckeridge, Cordell Neudorf

**Affiliations:** 1 Department of Health Management, Atlantic Veterinary College, University of Prince Edward Island, 550 University Avenue, Charlottetown, PE C1A 4P3 Canada; 2 Saskatchewan Health Quality Council, Atrium Building, Innovation Place, 241 – 111 Research Drive, Saskatoon, SK S7N 3R2 Canada; 3 College of Medicine, University of Saskatchewan, 107 Wiggins Road, Saskatoon, SK S7N 5E5 Canada; 4 Institute of Health Policy, Management and Evaluation, University of Toronto, Health Sciences Building 155 College Street, Suite 425, Toronto, ON M5T 3M6 Canada; 5 Institute for Clinical Evaluative Sciences, 2075 Bayview Ave., Toronto, ON M4N 3M5 Canada; 6 Centre for Addiction and Mental Health, 1001 Queen Street West, Toronto, ON M6J 1H4 Canada; 7 Saskatoon Health Region, #101–310 Idylwyld Drive North, Saskatoon, SK S7L 0Z2 Canada; 8 Department of Epidemiology, Biostatistics, and Occupational Health, McGill University, Purvis Hall 1020 Pine Avenue West, Montreal, QC H3A 1A2 Canada

**Keywords:** Mental health and addictions, High-cost users, Social determinants of health, Santé mentale et toxicomanie, Usagers à coût élevé, Déterminants sociaux de la santé

## Abstract

**Objective:**

A small proportion of the population accounts for the majority of healthcare costs. Mental health and addiction (MHA) patients are consistently high-cost. We aimed to delineate factors amenable to public health action that may reduce high-cost use among a cohort of MHA clients in Saskatoon, Saskatchewan.

**Methods:**

We conducted a population-based retrospective cohort study. Administrative health data from fiscal years (FY) 2009–2015, linked at the individual level, were analyzed (*n* = 129,932). The outcome of interest was ≥ 90th percentile of costs for each year under study (‘persistent high-cost use’). Descriptive analyses were followed by logistic regression modelling; the latter excluded long-term care residents.

**Results:**

The average healthcare cost among study cohort members in FY 2009 was ~ $2300; for high-cost users it was ~ $19,000. Individuals with unstable housing and hospitalization(s) had increased risk of persistent high-cost use; both of these effects were more pronounced as comorbidities increased. Patients with schizophrenia, particularly those under 50 years old, had increased probability of persistent high-cost use. The probability of persistent high-cost use decreased with good connection to a primary care provider; this effect was more pronounced as the number of mental health conditions increased.

**Conclusion:**

Despite constituting only 5% of the study cohort, persistent high-cost MHA clients (*n* = 6455) accounted for ~ 35% of total costs. Efforts to reduce high-cost use should focus on reduction of multimorbidity, connection to a primary care provider (particularly for those with more than one MHA), young patients with schizophrenia, and adequately addressing housing stability.

**Electronic supplementary material:**

The online version of this article (10.17269/s41997-018-0101-2) contains supplementary material, which is available to authorized users.

## Résumé

**Objectif:**

Une faible proportion de la population compte pour la majorité des coûts des soins de santé. Les patients en santé mentale et en toxicomanie (SMT) engendrent systématiquement des coûts élevés. Nous avons cherché à délimiter les facteurs qui se prêteraient à des mesures de santé publique susceptibles de réduire l’utilisation à coût élevé dans une cohorte d’usagers en SMT de Saskatoon (Saskatchewan).

**Méthode:**

Nous avons mené une étude de cohorte populationnelle rétrospective. Les données administratives sur la santé des exercices 2009 à 2015, jumelées au niveau individuel, ont été analysées (*n* = 129 932). Le résultat qui nous intéressait était ≥ au 90^e^ centile des coûts pour chaque exercice à l’étude (« utilisation à coût élevé persistante »). Des analyses descriptives ont été suivies par des modèles de régression logistique; ces derniers ont exclu les résidents en soins de longue durée.

**Résultats:**

Le coût moyen des soins de santé chez les membres de la cohorte étudiée était d’environ 2300 $ durant l’exercice 2009; pour les utilisateurs à coût élevé, il était d’environ 19 000 $. Les personnes vivant dans des conditions de logement précaires et ayant été hospitalisées une ou plusieurs fois présentaient un risque accru d’utilisation à coût élevé persistante; les deux effets s’accentuaient avec la présence de comorbidités. Les patients schizophrènes, surtout ceux de moins de 50 ans, présentaient une probabilité accrue d’utilisation à coût élevé persistante. La probabilité d’utilisation à coût élevé persistante diminuait avec la qualité du lien vers un dispensateur de soins primaires; cet effet s’accentuait avec l’augmentation du nombre de troubles de santé mentale.

**Conclusion:**

Bien qu’ils n’aient constitué que 5 % de la cohorte à l’étude, les usagers en SMT faisant une utilisation à coût élevé persistante des soins de santé (*n* = 6455) comptaient pour ~ 35 % des coûts totaux. Les efforts pour réduire l’utilisation à coût élevé devraient se concentrer sur la réduction des comorbidités, sur le lien vers un dispensateur de soins primaires (surtout pour les patients présentant plus d’un problème de SMT) et sur les jeunes patients schizophrènes, et ils devraient suffisamment tenir compte de la précarité du logement.

## Introduction

Evidence has long demonstrated that a small proportion of the population (< 10%) accounts for the majority (50–70%) of total healthcare spending (Densen et al. 1959): individuals commonly referred to as ‘high-cost users’. As early as 1988, Taube et al. demonstrated that individuals with mental disorders comprised 9% of high-cost users in 1 year (i.e., those with ≥ 25 visits), but accounted for nearly half (49.2%) of total outpatient expense (Taube et al. 1988). In a Medicaid population study of high-cost users, Buck et al. (2003) showed that people using mental health and substance abuse services constituted 11% of all enrollees but accounted for approximately 30% of all high-cost users (Buck et al. 2003). Hunter et al. (2015) found that nearly half of high-cost users had a mental health condition (Hunter et al. 2015).

Three Canadian studies have focused on patients with high costs and mental health and addiction (MHA) issues. de Oliveira et al. demonstrated that high-cost MHA patients incur 30% more healthcare costs per capita compared to high-cost users with no mental health conditions (de Oliveira et al. 2016b); a subsequent study demonstrated that MHA high-cost patients (‘MHA high-cost’ patients defined as individuals for whom MHA services accounted for ≥ 50% of their total healthcare costs) had healthcare costs 40% higher than those with no MHA-related costs (de Oliveira et al. 2017a). Using a combination of mood, substance use, psychotic and anxiety disorders as the definition of mental illness, Hensel et al. found that rates of mental illness were 39.3% in the top 1% costliest users (compared to 21.3% in the lowest cost group) (Hensel et al. 2016).

The disease burden of mental health and addiction disorders is large; it is estimated that in any given year, one in five Canadians will experience a mental illness or addiction, and, by age 40, one in two will have, or have had, a mental illness (Lim et al. 2008; Ratnasingham et al. 2013; Smetanin et al. 2011).

Mental health and addiction patients can have complex care needs related to their illness, as well as deficits in their social determinants of health, such as housing, income, education and employment (Walker and Druss 2016). Among MHA high-cost users, persons diagnosed with schizophrenia are more often high-cost, largely driven by hospitalizations (de Oliveira et al. 2016a; Fortney et al. 2009; Hensel et al. 2016; Junghan and Brenner 2006; Robst 2012). Stable housing, particularly, has been demonstrated to not only decrease healthcare service use, which is directly related to cost, but also improve health outcomes overall for mental health and addiction patients (Charkhchi et al. 2018; Fitzpatrick-Lewis et al. 2011; Hwang et al. 2005; Kerman et al. 2018; Rog et al. 2014; Stergiopoulos et al. 2018). Recent studies demonstrated residential instability as a risk factor for high-cost healthcare use at an ecological level (Thavorn et al. 2017) in addition to psychiatric inpatient cost savings (Rudoler et al. 2018). Given these previous findings, the current study included an administrative database definition of unstable housing in order to understand this covariate at an individual level in the study population.

Using population-based administrative health databases, we conducted an exploratory study to delineate factors amenable to public health action to prevent persistent high-cost use among a cohort of mental health and addiction clients in Saskatoon, Saskatchewan, Canada. We included healthcare utilization/access measures, such as connection to a primary care provider, and social determinants of health measures, such as unstable housing. The large cohort under study makes use of individual-level data, as opposed to previous studies (Thavorn et al. 2017) which rely on neighbourhood/ecological predictors.

## Methods

Saskatchewan (population ~ 1.2 million) is a Canadian province with a central provincial health insurer. Health data are captured for the provincial population with the exception of residents covered under the federal government (~ 1%), specifically inmates of federal prisons, members of the Royal Canadian Mounted Police (prior to 2013), Indigenous persons receiving primary healthcare services on federal reserve, and Canadian Armed Forces (Downey et al. 2006). The study population, due to database availability, was limited to Saskatoon Health Region, the largest health region in the province (*n* = 360,000).

### Databases

Detailed descriptions of Saskatchewan administrative health databases are available elsewhere (Downey et al. 2006). All data were linked at the individual level using the same unique encrypted identifier. Demographic characteristics, location of residence and insurance coverage were extracted from the *Personal Health Registration System* (*PHRS*). Hospital data for the province of Saskatchewan, and submitted to the Canadian Institute for Health Information (CIHI), were extracted from the *Discharge Abstract Database* (*DAD*) and include all hospitalizations (including psychiatric hospitalizations) in the province. The International Classification of Diseases (ICD), 10th revision, Canadian Version (ICD-10-CA) was used in the DAD to record up to 25 diagnoses at admission, including the most responsible one. Data on physician services are contained in the *Medical Services Database*. Physicians paid on a fee-for-service basis submit billing claims to the provincial health ministry; a single diagnosis using a three-digit ICD-9 code is recorded on each claim. Salaried physicians are required to submit ‘dummy’ claims for administrative purposes (shadow billing) but compliance is low; therefore, a level of under-reporting for salaried physician claims will exist. According to a recent CIHI report, a ‘relatively small percentage of Saskatchewan physicians are compensated through salaried arrangements’ (Canadian Institute for Health Information 2008). Residents of long-term care facilities were defined according to the provincial *Resident Assessment Instrument-Minimum Dataset* (*RAI-MDS*) for long-term care facilities (RAI-LTC 2.0). The following datasets were available for Saskatoon Health Region only (*n* = 162,566 individuals): emergency department data recorded in *National Ambulatory Care Reporting System* (*NACRS*); the *Shared Client Index* (*SCI*), an identity management system used to manage patient contact information; and the Population and Public Health, Saskatoon Health Region, *Street Outreach Database*. A combination of frequent address changes in the *Shared Client Index* and individuals in the *Street Outreach Database* permitted an administrative database definition of ‘unstable housing’. *Total government healthcare costs* by publicly funded source (hospital, physician, prescription drug and long-term care) were provided by the Saskatchewan Ministry of Health. Data were linked at the individual level using a unique non-identifiable health services number generated by eHealth Saskatchewan.

### Study design

We conducted a retrospective population-based study using administrative health data. The study population included any individual with continuous provincial insurance coverage, resident of Saskatoon Health Region, ≥ 18 years old, alive as of April 1, 2009 (study baseline) and with at least one mental health or addiction-related International Classification of Disease Codes (ICD-9 or ICD-10) diagnosis in any database from April 1, 2003, to March 31, 2015, with the exception of dementia and dementia-related codes (Table S1). Total healthcare costs were calculated by unique individual for each fiscal year 2009–2015.

### Exposure

Using a combination of emergency department, physician and hospital data, mental health and addiction ICD-10 codes were grouped into the following: substance-related disorders; schizophrenia, delusional and non-organic psychotic disorders; mood/affective disorders; anxiety disorders; and selected disorders of adult personality and behaviour (Table S1).

### Outcome

The primary end-point was *persistent high-cost healthcare use* (≥ 90th percentile for each fiscal year 2009 to 2015). Using the costing methodology developed by Wodchis et al.*,* all costs were estimated and assigned to unique study individuals (Wodchis et al. 2013). Briefly, this methodology provides guidance on how to identify unit costs associated with individual healthcare utilization and how to combine these with utilization data from administrative databases, providing a measure of direct healthcare costs incurred by government. To account for death during the study period, each study subject’s total healthcare costs were divided by total number of days observed in the study.

High-cost status among cohort members was determined for each year of the study period using population proportional thresholds (Wodchis et al. 2016). As cost was shown to vary by individual over time, variables of ‘never high-cost’ (never ≥ 90th percentile for any year of the study period), ‘sometimes high cost’ (≥ 90th percentile in any one year) and ‘persistent high-cost’ (≥ 90th percentile for all years, including up to the point of death) were calculated. Total costs were a sum of mutually exclusive government costs for hospitalizations, prescription drugs, emergency department visits, physician services, and long-term care. Physician billing and prescription drug costs were direct totals payable by the provincial health insurer. Hospitalization costs were obtained using the Standard Cost of a Hospital Stay multiplied by the specific resource intensity weight (RIW) at the individual-level (Canadian Institute for Health Information (CIHI) 2015a, b). Long-term care (LTC) costs were defined using the actual government LTC expenditure provided by the Community Care Branch, Saskatchewan Ministry of Health, and calculated as a daily cost per bed. This cost was multiplied by the number of days an individual was resident in LTC. Study cohort costs were further defined as an annual weighted cost, weighted based on the number of days the individual was alive per study year. All yearly costs were adjusted for inflation to FY 2015.

### Covariates

Variables included were as follows: age, sex, urban/rural location of residence, neighbourhood-level income quintile, death, number of mental health conditions, comorbid conditions, Usual Provider Continuity (UPC) index, unstable housing, and healthcare utilization measures. All covariates were defined at the point of occurrence (such as death), otherwise, as of study baseline April 1, 2009, permitting a temporal association between exposures (covariates) and persistent high-cost use (outcome).

The UPC index developed by CIHI was used to indicate the degree to which an individual is connected to a primary care provider. Each physician in the province is assigned a unique number. The UPC is a proportion—for each study cohort member, the total number of physician visits to the same general practitioners’ number is divided by the total number of physician visits to all general practitioners over the same time period. For regression modelling, a dichotomous variable of the UPC index score was created using CIHI’s categorization of 0 to < 0.75 (poorly connected) versus 0.75+ (well connected) (Canadian Institute for Health Information 2016).

Neighbourhood-level income quintile was based on dissemination area average household income values from public-use Statistics Canada census files using the postal code methodology developed by the Institut National de Santé Publique du Québec (INSPQ 2007). As hospital stays are known to drive healthcare costs, ‘hospitalization’ was defined as ever having an acute in-patient stay prior to the costing observation period. Unstable housing was defined as street involvement (street outreach client, *n* = 967), or ≥ 4 address changes in a 12-month period from 2003 to 2009 (*n* = 299).

All hospitalizations, emergency department and physician visits (specialty and general practice) from April 1, 2003, to March 31, 2009, were summed by mental health and addiction diagnostic category. As a large proportion of the study cohort had more than one mental health condition (37%), ‘primary mental health condition’ was assigned as the most frequent MHA diagnostic code(s) across physician, hospitalization and emergency department in the study period.

Comorbidities were defined using validated case definitions for chronic disease conditions from the Canadian Chronic Disease Surveillance System (CCDSS): (1) chronic obstructive pulmonary disease (COPD), (2) congestive heart failure (CHF), (3) asthma, (4) diabetes, and (5) coronary artery disease (CAD) (Public Health Agency of Canada 2003; Feely et al. 2017). For regression modelling, the total number of comorbid conditions was used (0 to 5). Death data were obtained using a ‘verified death file’ (combination of death data from various administrative health databases) created by the Saskatchewan Health Quality Council for research purposes.

### Statistical analyses

Following univariate and bivariate analyses, multivariate regression modelling was used to delineate factors associated with persistent high-cost use. Classification and regression tree (CART) modelling facilitated a visual understanding of variables contributing most to persistent high-cost use; subsequent logistic regression modelling, probit link, quantified the relationship between persistent high-cost use and significant co-variates. In logistic modelling, missing values (income quintile and location of residence) were coded as ‘missing’. Sensitivity analyses with and without missing data were conducted.

All long-term care residents (*n* = 5435; 4.2% of cohort) were excluded from the logistic modelling. Approximately 36% of persistent high-cost users were residents of a LTC facility. As we aimed to delineate factors amenable to public health action to prevent persistent high-cost use, it was considered reasonable to exclude these study subjects. It was assumed that individuals in LTC facilities met the standardized criteria for requiring their higher level of care and their reason for persistent high-cost use was known.

All analyses were conducted using SAS© Enterprise Guide version 7.1 (SAS Institute Inc. 2017).

The study proposal underwent ethical review and approval by the University of Saskatchewan Biomedical Research Ethics Board, the University of Prince Edward Island Research Ethics Board and the Health Canada/Public Health Agency of Canada Research Ethics Board for research involving humans.

## Results

**Fig. 1:**
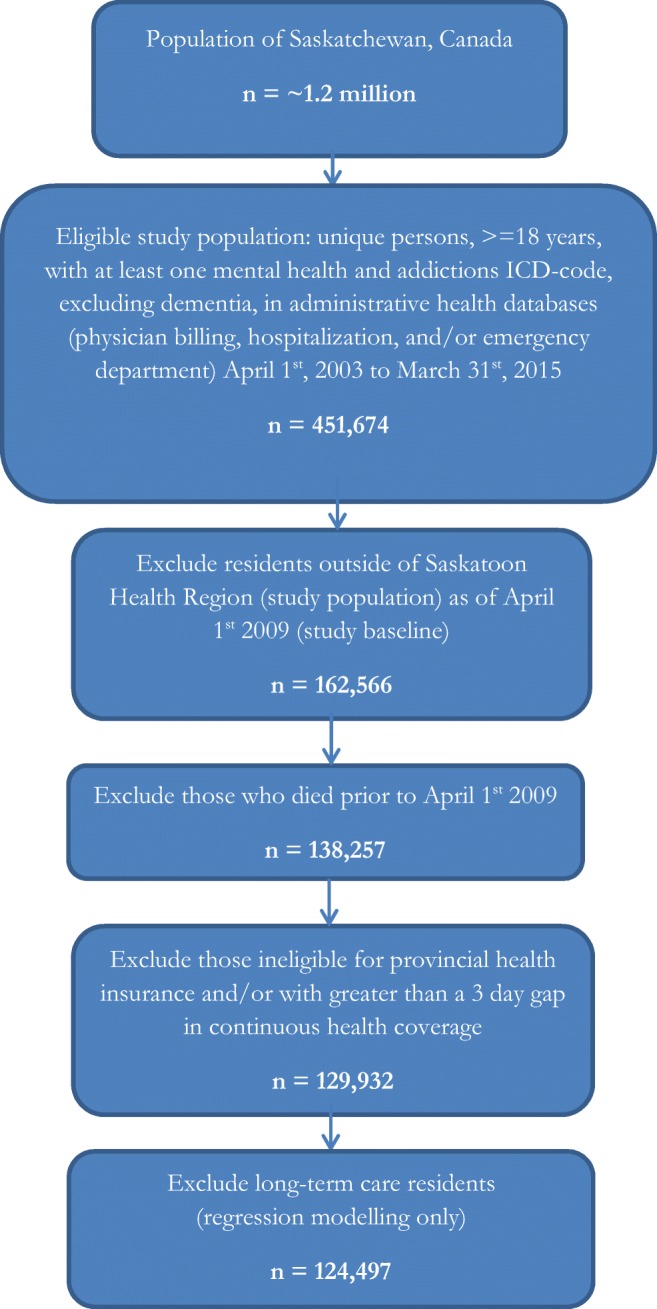
Study cohort inclusion/exclusion criteria

A total of 129,932 unique individuals eligible for provincial health insurance, resident of Saskatoon Health Region (SHR) and alive as of the study baseline date (April 1, 2009) were identified as belonging to the study cohort (Fig. 1). Of these, the majority (55%) were ‘never high-cost’; persistent high-cost users accounted for 5% of the study cohort. Cohort characteristics at study baseline are detailed in Table 1.

**Table 1:** Descriptive statistics, mental health and addiction study cohort, Saskatoon Health Region, Saskatchewan (*n* = 129,932)

Covariates (*n*) (%)/(mean) (SD)	Never high-cost *n* = 71,834 *n* (%) or mean (SD)	Sometimes high-cost *n* = 51,643 *n* (%) or mean (SD)	Persistent high-cost *n* = 6455 *n* (%) or mean (SD)
Mean age, years (SD)	42 (15)	51 (19)	68 (17)
Sex			
Female (57%)	37,599 (52%)	33,020 (64%)	3639 (56%)
Male (43%)	34,235 (48%)	18,623 (36%)	2816 (44%)
Location of residence			
Urban (84%)	60,668 (85%)	42,799 (83%)	5363 (83%)
Rural (15%)	10,285 (14%)	8281 (16%)	1050 (16%)
Missing* (1%)	881 (1%)	563 (1%)	42 (1%)
Primary mental health diagnosis			
Substance-related disorders (15%)	11,458 (16%)	7067 (14%)	676 (10%)
Schizophrenic disorders (5%)	1025 (1%)	3126 (6%)	1707 (26%)
Mood/affective disorders (27%)	19,594 (27%)	15,376 (30%)	1810 (28%)
Anxiety disorders (52%)	39,524 (55%)	25,928 (50%)	2235 (35%)
Disorders of adult personality (1%)	233 (1%)	146 (1%)	27 (1%)
Number of mental health conditions (mean; SD)	1.4 (0.6)	1.6 (0.8)	2.0 (1.0)
Physician visits per year (mean; SD)	5.5 (5.4)	10.2 (8.6)	24.7 (18.2)
Emergency department visits per year (mean; SD)	0.2 (0.4)	0.4 (0.9)	1.1 (2.1)
Number of hospitalizations per year (mean; SD)	0.1 (0.2)	0.3 (0.3)	0.7 (0.9)
At least 1 psychiatrist visit (16%)	9222 (13%)	8806 (17%)	2294 (36%)
Mean healthcare cost per year ($; SD)	$557 ($745)	$3242 ($7467)	$28,320 ($35,003)
Income quintile			
1 (least affluent) (20%)	13,799 (19%)	11,180 (22%)	1587 (25%)
2 (18%)	12,633 (18%)	9033 (18%)	1165 (18%)
3 (20%)	14,456 (20%)	10,260 (20%)	1263 (20%)
4 (18%)	12,927 (18%)	9012 (17%)	1013 (16%)
5 (most affluent) (18%)	13,113 (18%)	8833 (17%)	1028 (16%)
Missing* (6%)	4906 (7%)	3325 (6%)	399 (5%)
Unstable housing (1%)	412 (1%)	691 (1%)	163 (3%)
Connection to primary care doctor			
Well connected (43%)	27,196 (38%)	25,202 (49%)	3056 (47%)
Not well connected (57%)	44,638 (62%)	26,441 (51%)	3399 (53%)
Select comorbid conditions			
Diabetes (12%)	4267 (6%)	8635 (17%)	2562 (40%)
Chronic obstructive pulmonary disease (9%)	3067 (4%)	6662 (13%)	2147 (33%)
Congestive heart failure (7%)	905 (1%)	5484 (11%)	2645 (41%)
Coronary artery disease (11%)	2346 (3%)	9535 (19%)	2843 (44%)
Asthma (9%)	4990 (7%)	5508 (11%)	1097 (17%)
Died during study period (FY 2009–2015) (7%)	686 (1.0%)	4249 (8%)	3515 (55%)
Long-term care resident			
Yes** (4%)	13 (0.02%)	3074 (6%)	2348 (36%)
No (96%)	71,821 (99.8%)	48,569 (94%)	4107 (64%)

*Modelled categorically as ‘missing’ in regression modelling

**Excluded from regression modelling

Compared to never high-cost, persistent high-cost users had higher proportions of females, schizophrenia diagnosis, unstable housing, and deaths. Approximately 30% of high-cost users in FY 2009 went on to be high-cost in subsequent years (data not shown). Persistent high-cost users were less likely to have a primary mental health diagnosis of anxiety and more likely to have a diagnosis of schizophrenia, delusional and non-organic psychotic disorders (Table 1). Individuals with a primary mental health condition of schizophrenia had total healthcare costs ~ five times higher, and nearly twice the average number of hospitalizations and physician visits, compared to all other MHA diagnoses (Table 2).

**Table 2:** Average annual cost and healthcare utilization by mental health diagnosis, Saskatoon Health Region, FY 2009–2015 (*n* = 129,932)

Primary mental health condition	*n*	Annual healthcare cost Mean (SD); median	Annual physician visits Mean (SD)	Annual emergency department visits Mean (SD)	Annual hospitalizations Mean (SD)
Substance-related disorders	19,201	$1905 ($5253); $502	7 (9)	0.4 (1.4)	0.2 (0.3)
Schizophrenia	5858	$12,488 ($16,080); $5138	15 (13)	0.5 (1.1)	0.4 (0.7)
Mood/affective disorders	36,780	$2251 ($5346); $847	9 (9)	0.3 (0.7)	0.2 (0.3)
Anxiety disorders	67,687	$1765 ($4195); $647	8 (8)	0.3 (0.6)	0.2 (0.3)
Disorders of adult personality	406	$2446 ($6078); $646	9 (12)	0.4 (1.3)	0.2 (0.4)

**Fig. 2:**
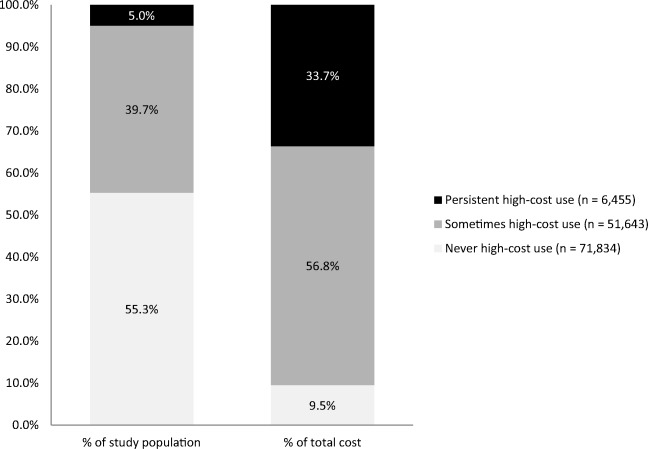
Mental health and addiction study cohort by total yearly cost, Saskatoon Health Region, Saskatchewan, FY 2009–2015 (*n* = 129,932)

Despite comprising only 5% of the population, persistent high-cost users (*n* = 6455) accounted for nearly 35% of total costs (FY 2009). Never high-cost users (*n* = 71,834) accounted for over half of the study population but less than 10% of costs; sometimes high-cost users (*n* = 51,643) accounted for approximately 40% of the study population and 57% of the costs (Fig. 2).

Multivariable logistic regression identified predictors statistically significantly associated with persistent high-cost use. Compared to the logit link, a probit link provided a better fitting model, common in studies using healthcare cost as an outcome and susceptible to heteroscedasticity (Basu et al. 2006). Relevant two-way interactions and one three-way interaction term were retained in the final model as they improved model performance. Age, sex, and primary mental health condition interacted (*p* = 0.005); patients with schizophrenia, particularly those under 50 years old, had increased probability of persistent high-cost use; females with anxiety had higher probability of persistent high-cost use compared to males. Neighbourhood-level income quintile, when taken into account with all other predictors, was not statistically significantly associated with persistent high-cost use (*p* = 0.3). Several covariates (connection to a primary care provider, number of mental health conditions, number of comorbid conditions) interacted with ‘died during study period’. All interaction terms demonstrated increased risk for persistent high-cost use when study subjects died during the observation period. This occurred despite accounting for number of days alive in the study period when assigning costs and excluding long-term care residents in the modelling. Sensitivity analyses including and excluding patients who died during the study period did not differ in overall findings/conclusion.

A number of statistically significant two-way interactions occurred. Connection to a primary care provider was protective of persistent high-cost use in general, but particularly for individuals with multiple mental health conditions (*p* = 0.001). Unstable housing increased the probability of persistent high-cost use in general, but this effect was more pronounced with 2+ comorbid conditions (*p* = 0.02). Similarly, hospitalization increased the probability of persistent high-cost use, but particularly for those with two or more comorbidities (*p* = 0.01) (Table 3). The model showed evidence of good fit.

**Table 3:** Regression modelling, binary distribution, probit link, comparing persistent high-cost with not persistently high-cost use, mental health and addiction cohort, excluding long-term care residents, Saskatoon Health Region, FY2009–2015 (*n* = 124,497)

Predictor	Estimate	*SE*	*p* value
Age			0.002
< 49 years	(reference)		
≥ 50 years	0.14	0.05	
Sex			0.561
Male	(reference)		
Female	0.03	0.05	
Mental health condition			< 0.001
Anxiety disorders	(reference)		
Mood/affective disorders	0.25	0.05	
Schizophrenic disorders	0.94	0.07	
Substance-related disorders	0.13	0.06	
Housing			< 0.001
Stable housing	(reference)		
Unstable housing	0.39	0.07	
Comorbid conditions			< 0.001
None	(reference)		
1	0.43	0.04	
2 or more	0.92	0.05	
Connection to a primary care provider			0.002
Not connected	(reference)		
Well connected	0.09	0.03	
Number of mental health conditions			< 0.001
1	(reference)		
2 or more	0.29	0.03	
Hospitalization(s)			< 0.001
No	(reference)		
Yes	0.54	0.03	
Died during study period			< 0.001
No	(reference)		
Yes	1.95	0.05	
Connection to a primary care provider*, number of mental health conditions			0.001
Well connected, 2 or more	0.12	0.04	
Housing*, comorbid conditions			0.023
Unstable housing, 1 comorbid condition	− 0.10	0.12	
Unstable housing, 2 or more comorbid conditions	− 0.35	0.13	
Comorbid conditions*, hospitalization(s)			0.010
1 comorbid, hospitalized	− 0.05	0.05	
2 or more, hospitalized	0.12	0.05	
Comorbid conditions*, died during study period			< 0.001
1, died	− 0.23	0.06	
2 or more, died	− 0.38	0.05	
Number of mental health conditions*, died during study period			< 0.001
2 or more, died	− 0.33	0.05	
Connection to a primary care provider*, died during study period			< 0.001
Well connected, died	− 0.64	0.04	
Age*, mental health condition			< 0.001
≥ 50 years, mood	− 0.14	0.06	
≥ 50 years, schizophrenia	− 0.73	0.10	
≥ 50 years, substance-related	− 0.19	0.07	
Age*, sex			0.993
≥ 50 years, female	0.001	0.06	
Sex*, mental health condition			0.101
Female, mood	− 0.12	0.06	
Female, schizophrenia	0.06	0.11	
Female, substance-related	− 0.12	0.08	
Age*, sex*, mental health condition			0.005
≥ 50 years, female, mood	0.18	0.08	
≥ 50 years, female, schizophrenia	− 0.30	0.14	
≥ 50 years, female, substance-related	0.08	0.11	
*Intercept*	− 3.09	0.05	

Area under the curve = 0.90; Hosmer and Lemeshow goodness-of-fit: *χ*^2^ = 11.1; *df* = 8; *p* = 0.2

*Disorders of adult personality are included with mood/affective disorders due to small cell sizes (< 5 when stratified)

All pairwise comparisons in interaction terms were adjusted for multiple comparisons (Bonferroni’s method) (Table S2).

## Discussion

Average healthcare costs among study cohort members in FY 2009 were approximately $2300; those among high-cost users were nearly $19,000. Despite making up less than 5% of the study population, persistent high-cost mental health and addiction clients in SHR accounted for nearly 35% of total SHR costs. In multivariable logistic regression, even after accounting for other potentially confounding factors, unstable housing was found to increase the probability of persistent high-cost use in the study population; a higher probability occurred if the individual had other underlying health conditions. A recent study in Ontario by Thavorn et al. similarly demonstrated, at an ecologic level, the association between residential instability and multimorbidity in high costs (Thavorn et al. 2017).

Homelessness is known to be associated with increased healthcare utilization, poor health status, health inequities, and mental health conditions (Aldridge and Kelley 2015). The complexities of co-occurrence of mental illness, chronic medical conditions, and housing status need to be taken into account when conceptualizing and addressing multimorbidity (Walker and Druss 2016). Individuals with complex MHA issues may cope with co-existing conditions (such as diabetes or heart disease) less well than individuals without mental illness (Prior et al. 2018). The positive effects of stable housing on health outcomes have been well documented—resulting in fewer communicable diseases, injuries, better chronic disease management/prevention, and improved psycho-social well-being (Fitzpatrick-Lewis et al. 2011).

Policy makers, concerned with healthcare cost containment, are reasonable to focus on the small groups of patients consuming the majority of resources. However, ‘high-cost users’ are not a homogenous group. Even when focusing on a particular patient group that is known to be high-cost, in this case mental health and addictions, and further refining the population of interest into persistently high-cost over many years, heterogeneity in the patient population still occurred. Despite this potentially heterogeneous study population, and taking into account other, potentially confounding, factors, such as healthcare utilization and demographic factors, unstable housing remained a significant risk factor for persistent high-cost use.

In terms of public health actions to address housing stability, a recent review of the evidence concluded policy makers should consider providing permanent supportive housing for homeless/disabled MHA patients (Rog et al. 2014). The provision of a stable home, particularly for individuals with MHA and/or complex care needs, not only can decrease healthcare utilization/costs, but can improve health outcomes in a vulnerable population (Dieterich et al. 2017; Fitzpatrick-Lewis et al. 2011; Hwang et al. 2005; Poremski et al. 2016). It may also be reasonable to consider shifting resources, from costly acute care for mental health and addiction patients to providing better models of care in the community and connection to a primary care provider, particularly where multiple comorbid conditions and housing instability are present. A recent Canadian study demonstrated that community-based, coordinated access to care programs provide improved health outcomes, cost-effectively, among a cohort of homeless adults with mental health needs (Stergiopoulos et al. 2018). This points to the potential to identify mental health clients with unstable housing and comorbid conditions earlier and offer ‘Housing First’ or other supportive housing services, potentially preventing worsening of both their mental health and their other comorbidities and increasing their ability to cope (Chrystal et al. 2015; Poremski et al. 2016).

From published estimates (de Oliveira et al. 2017b, 2016c; Knapp et al. 2004), we know that schizophrenia and eating disorders are among the most expensive mental health and addiction diagnostic categories. In the current study, eating disorders are not delineated due to privacy considerations of small numbers in Saskatchewan (< 5 individuals per year); schizophrenic disorders were the most expensive diagnostic category.

As ‘high-cost use’ changes over time, even within the same individual, categories of persistent, sometimes, and never high-cost were created. Studies describing high-cost use as a snapshot in time may be combining potentially heterogeneous categories of individuals. As noted by Wodchis et al., one third of high-cost users in Ontario were found to be persistently high-cost in the subsequent 2-year study follow-up (Wodchis et al. 2016); a similar proportion was found in the current study.

Not surprisingly, we found hospitalization increased the probability of persistent high-cost use. It is difficult to quantify ‘avoidable’ hospitalizations in administrative health data; however, a previous study in New Zealand estimated one in three hospitalizations was avoidable (Jackson and Tobias 2001).

We found that having a good connection to a primary care provider decreased the probability of being a high-cost user. Hospitalization is the costliest form of care in the healthcare system. Efforts to reduce persistent high-cost use could assess whether or not adequate community-based care could offset the costs associated with hospitalization(s).

Interestingly, despite accounting for number of days alive in the study period and excluding long-term care residents in the modelling, having died during the study period continued to be a significant risk factor for persistent high-cost use. Future studies could examine end-of-life costs, particularly if certain interventions can improve a patient’s experience while at the same time reducing costs.

This study has several limitations, some inherent to administrative health databases. By focusing on administrative health databases, we could not account for all potential confounders; for example, food insecurity (Tarasuk et al. 2015) has been found to be associated with high-cost use but not available for analysis. Community-based mental health service provision (counselling, treatment centres, others) may be associated with persistent high-cost use but data on these services were not available for analysis. Our study focused on cost; however, not all healthcare costs were measured, such as home care, ambulatory care, out-of-pocket healthcare costs, such as prescription medication costs covered by private insurance, travel costs (air transfers and ground ambulance) and all indirect costs (caregiving or lost wages). Misclassification likely occurred when categorizing individuals. For example, individuals with zero healthcare use and unstable housing would not be counted, underestimating the number of people with unstable housing. National data quality processes are in place for hospitalization and emergency department data; however, physician billing data can have diagnostic limitations. Despite this, reliability/validity has been found to be fairly good (Lix et al. 2013).

Results from this study demonstrate that efforts to reduce persistent high-cost use among a cohort of mental health and addiction clients should focus on multimorbidity, connection to a primary care provider (particularly for those with more than one mental health condition), young patients with schizophrenia, and adequately addressing housing stability.

## Electronic supplementary material

ESM 1 (DOCX 13.7 kb)

ESM 2 (DOCX 16.8 kb)
