# Supplementary material for: The effect of socio-demographic factors on mental health and addiction high-cost use: a retrospective, population-based study in Saskatchewan
Source: Can J Public Health. 2018 Jun 28;109(5-6):810–20. doi: 10.17269/s41997-018-0101-2 (PMC6267642; doi:10.17269/s41997-018-0101-2)
Supplement: Supplementary file 1 — (DOCX 13.7 kb) [file 41997_2018_101_MOESM1_ESM.docx]

**Table S1:** Mental health and addictions ICD codes by condition

| **Condition*** | **ICD-9** | **ICD-10** |
| --- | --- | --- |
| Substance related disorders | 291, 292; 303-305 | F10-F19; F55 |
| Schizophrenia, schizotypal, delusional and other non-mood psychotic disorders | 295, 297, 298 | F20-F25; F28, F29 |
| Mood/affective disorders | 296, 311 | F30-F34; F39 |
| Anxiety disorders | 300, 308, 309 | F40-F48; F93 |
| Disorders of adult personality | 301 | F60-F69 |

^* Individuals >=18 years, with one or more physician claims with the relevant ICD-9 diagnostic code, or, one ICD-10-CA hospital discharge in any diagnosis field(s)^
