# Supplementary material for: The effect of socio-demographic factors on mental health and addiction high-cost use: a retrospective, population-based study in Saskatchewan
Source: Can J Public Health. 2018 Jun 28;109(5-6):810–20. doi: 10.17269/s41997-018-0101-2 (PMC6267642; doi:10.17269/s41997-018-0101-2)
Supplement: Supplementary file 2 — (DOCX 16.8 kb) [file 41997_2018_101_MOESM2_ESM.docx]

**Table S2:** **Probability estimates of interaction terms, regression model mental health and addictions cohort, excluding long-term care residents, Saskatoon Health Region, FY2009-2015 (n = 124,497)**

| **Predictor** | **Estimate** | **95% CI** | **Group*** |
| --- | --- | --- | --- |
| Male, < 49 years, anxiety | 0.001 | 0.0007-0.001 | C |
| Male, < 49 years, mood/affective disorders | 0.002 | 0.002-0.003 | B |
| Male, < 49 years, schizophrenia | 0.016 | 0.011-0.022 | A |
| Male, < 49 years, substance-related disorders | 0.002 | 0.001-0.002 | B, C |
| Female, < 49 years, anxiety | 0.001 | 0.0008-0.001 | C |
| Female, < 49 years, mood/affective disorders | 0.002 | 0.001-0.002 | B |
| Female, < 49 years, schizophrenia | 0.019 | 0.013-0.029 | A |
| Female, < 49 years, substance-related disorders | 0.001 | 0.001-0.002 | B, C |
| Male, > = 50 years, anxiety | 0.002 | 0.001-0.002 | B, C |
| Male, > = 50 years, mood/affective disorders | 0.003 | 0.002-0.004 | A |
| Male, > = 50 years, schizophrenia | 0.002 | 0.001-0.002 | A |
| Male, > = 50 years, substance-related disorders | 0.001 | 0.001-0.002 | C |
| Female, > = 50 years, anxiety | 0.002 | 0.001-0.002 | B |
| Female, > = 50 years, mood/affective disorders | 0.003 | 0.002-0.004 | B |
| Female, > = 50 years, schizophrenia | 0.002 | 0.001-0.002 | B |
| Female, > = 50 years, substance-related | 0.001 | 0.001-0.002 | B |
| Not connected to primary care provider*One MH condition | 0.001 | 0.0007-0.001 | A |
| Connected to primary care provider*One MH condition | 0.001 | 0.001-0.002 | B |
| Not connected to primary care provider*Two or more mental health conditions | 0.003 | 0.002-0.003 | C |
| Connected to primary care provider*Two or more mental health conditions | 0.005 | 0.004-0.006 | D |
| Unstable housing, no comorbid conditions | 0.003 | 0.002-0.005 | B |
| Unstable housing, one comorbid condition | 0.009 | 0.005-0.015 | A, B |
| Unstable housing, two or more comorbid conditions | 0.016 | 0.009-0.029 | A |
| Stable housing, no comorbid conditions | 0.001 | 0.007-0.001 | C |
| Stable housing, one comorbid condition | 0.004 | 0.003-0.005 | B |
| Stable housing, two or more comorbid conditions | 0.016 | 0.009-0.029 | A |
| Hospitalized, no comorbid conditions | 0.005 | 0.002-0.007 | D |
| Hospitalized, one comorbid condition | 0.015 | 0.012-0.018 | B |
| Hospitalized, two or more comorbid conditions | 0.065 | 0.054-0.077 | A |
| Not hospitalized, no comorbid conditions | 0.001 | 0.0007-0.001 | E |
| Not hospitalized, one comorbid condition | 0.004 | 0.003-0.005 | C |
| Not hospitalized, two or more comorbid conditions | 0.015 | 0.011-0.018 | B |

^*Adjusted for multiple comparisons (Bonferroni method); Estimates with the same letter are not significantly different^
